# Molybdenum Exposure and Semen Quality: How Robust Is the Evidence of an Effect?

**DOI:** 10.1289/ehp.0900922

**Published:** 2009-09

**Authors:** Tom Sorahan, Frank M. Sullivan

**Affiliations:** Institute of Occupational and Environmental Medicine, University of Birmingham, Birmingham, United Kingdom, Email: T.M.Sorahan@bham.ac.uk; Toxicologist, Brighton, United Kingdom

Meeker et al. (2008) reported on associations between blood levels of nine different metals and four aspects of semen quality found in 219 volunteers attending two infertility clinics. Based on the title of their article, it seems that the authors claimed to have found evidence that molybdenum is a male reproductive toxicant. But how robust is the evidence? Meeker et al. (2008) had no primary prior hypotheses, and their study was essentially a “fishing expedition” involving many statistical tests of many possible associations. The authors (Meeker et al. 2008) did note that “some [findings] may be chance findings because of the number of comparisons that were made.” A much stronger statement is appropriate, namely, that it is very likely that some of the findings were chance findings.

The overwhelming majority of study subjects (70%) had blood Mo levels below the study limit of detection of 1 μg/L. If a more sensitive assay method had been used [e.g., the method of Case et al. (2001) with a detection limit of 0.06 μg/L], then a better grouping of subjects could have been used in the data analyses. We also believe the authors made an inappropriate analytical decision. In Table 3 of their article, Meeker et al. (2008) summarized analyses in which dose-dependent associations for each metal were considered in turn while adjusting for age and current smoking. The analysis of associations between Mo concentrations and semen motility were based on data from 147 subjects. The assumption appears to be that 72 (219 – 147) subjects had no useful information to provide on the Mo/sperm motility issue. It would have been more scientifically rigorous to allow the analysis of each semen parameter to make use of data from all study subjects, and not just for the sake of completeness. The current approach has introduced bias into the analysis, because different standards are applied to cases [subjects with the adverse health outcome of interest (e.g. poor sperm concentration)] and controls or referents (subjects without the adverse health outcome). To be concrete, in the analyses of sperm concentrations, cases were allowed to have poor sperm motility, whereas controls were not. The effect of this, in practice, is to load the controls with subjects on high incomes who have never smoked. A more standard analysis involving all study subjects in the analysis of each aspect of sperm quality should have been carried out. Such an analysis would likely have provided very different results to the subset analyses they reported.

In commenting on the linear regression analyses shown in their Table 4, Meeker et al. (2008) stated that the Mo groups “were associated with suggestive decreasing trends in sperm concentration and morphology.” However, examination of the table shows that neither of the regression coefficients was significantly different from zero (i.e., the blood Mo level had no significant influence on any of the semen quality parameters).

Additional studies would be needed to investigate possible effects in semen quality based on much larger population samples containing a wider range of blood Mo levels.
